# Macrophage invasion contributes to degeneration of stria vascularis in Pendred syndrome mouse model

**DOI:** 10.1186/1741-7015-4-37

**Published:** 2006-12-22

**Authors:** Sairam V Jabba, Alisha Oelke, Ruchira Singh, Rajanikanth J Maganti, Sherry Fleming, Susan M Wall, Lorraine A Everett, Eric D Green, Philine Wangemann

**Affiliations:** 1 Anatomy & Physiology Department, Kansas State University, Manhattan KS, 66506, USA; 2 Division of Biology, Kansas State University, Manhattan KS, 66506, USA; 3 Department Medicine, Renal Division, Emory University, School of Medicine, Atlanta, Georgia, USA; 4 Genome Technology Branch, National Human Genome Research Institute, National Institutes of Health, Bethesda, Maryland, USA

## Abstract

**Background:**

Pendred syndrome, an autosomal-recessive disorder characterized by deafness and goiter, is caused by a mutation of *SLC26A4*, which codes for the anion exchanger pendrin. We investigated the relationship between pendrin expression and deafness using mice that have (*Slc26a4*^+/+ ^or *Slc26a4*^+/-^) or lack (*Slc26a4*^-/-^) a complete *Slc26a4 *gene. Previously, we reported that stria vascularis of adult *Slc26a4*^-/- ^mice is hyperpigmented and that marginal cells appear disorganized. Here we determine the time course of hyperpigmentation and marginal cell disorganization, and test the hypothesis that inflammation contributes to this tissue degeneration.

**Methods:**

*Slc26a4*^-/- ^and age-matched control (*Slc26a4*^+/+ ^or *Slc26a4*^+/-^) mice were studied at four postnatal (P) developmental stages: before and after the age that marks the onset of hearing (P10 and P15, respectively), after weaning (P28-41) and adult (P74-170). Degeneration and hyperpigmentation stria vascularis was evaluated by confocal microscopy. Gene expression in stria vascularis was analyzed by microarray and quantitative RT-PCR. In addition, the expression of a select group of genes was quantified in spiral ligament, spleen and liver to evaluate whether expression changes seen in stria vascularis are specific for stria vascularis or systemic in nature.

**Results:**

Degeneration of stria vascularis defined as hyperpigmentation and marginal cells disorganization was not seen at P10 or P15, but occurred after weaning and was associated with staining for *CD68*, a marker for macrophages. Marginal cells in *Slc26a4*^-/-^, however, had a larger apical surface area at P10 and P15. No difference in the expression of *Lyzs*, *C3 *and *Cd45 *was found in stria vascularis of P15 *Slc26a4*^+/- ^and *Slc26a4*^-/- ^mice. However, differences in expression were found after weaning and in adult mice. No difference in the expression of markers for acute inflammation, including *Il1a*, *Il6*, *Il12a*, *Nos2 *and *Nos3 *were found at P15, after weaning or in adults. The expression of macrophage markers including *Ptprc *(= *Cd45*), *Cd68*, *Cd83*, *Lyzs*, *Lgals3 *(= *Mac2 *antigen), *Msr2*, Cathepsins B, S, and K (*Ctsb, Ctss, Ctsk*) and complement components *C1r*, *C3 *and *C4 *was significantly increased in stria vascularis of adult *Slc26a4*^-/- ^mice compared to *Slc26a4*^+/+ ^mice. Expression of macrophage markers *Cd45 *and *Cd84 *and complement components *C1r *and *C3 *was increased in stria vascularis but not in spiral ligament, liver or spleen of *Slc26a4*^-/- ^compared to *Slc26a4*^+/- ^mice. The expression of *Lyzs *was increased in stria vascularis and spiral ligament but not in liver or spleen.

**Conclusion:**

The data demonstrate that hyperpigmentation of stria vascularis and marginal cell reorganization in *Slc26a4*^-/- ^mice occur after weaning, coinciding with an invasion of macrophages. The data suggest that macrophage invasion contributes to tissue degeneration in stria vascularis, and that macrophage invasion is restricted to stria vascularis and is not systemic in nature. The delayed onset of degeneration of stria vascularis suggests that a window of opportunity exists to restore/preserve hearing in mice and therefore possibly in humans suffering from Pendred syndrome.

## Background

Pendred syndrome is an autosomal recessive disorder that is characterized by profound sensorineural deafness, abnormal iodide transport across the thyroid follicular epithelium and an enlarged vestibular aqueduct [1,2]. It is an important condition as it accounts for 1–10% of all cases of hereditary deafness [3]. Pendred syndrome is caused by mutations of the gene SLC26A4, which codes for the protein pendrin [4]. Hearing loss in Pendred syndrome develops in most cases prelingually, which implies that pendrin is not essential for hearing but that a defective pendrin protein causes hearing loss via a secondary mechanism [3,5]. Most Pendred syndrome patients are euthyroid, although the abnormal iodide transport in the thyroid affects the incorporation of iodide into thyroglobulin [6,7]. It is conceivable that the observed thyroid hyperplasia (goiter), which generally develops around puberty, ensures normal levels of thyroid hormone [2].

Pendrin is a Na^+^-independent exchanger for anions such as Cl^-^, I^-^, HCO_3 _^- ^and formate [8-10]. Pendrin is expressed in the inner ear, thyroid, kidney, mammary gland, uterus, testes, and placenta [6,11-17]. In the thyroid, pendrin is expressed on the apical side of the thyrocytes and mediates Cl^-^/I^- ^exchange. In the kidney, pendrin is expressed on the apical side of the non-A, non-B intercalated cells, cytoplasmic regions of the type B intercalated cells of cortical collecting tubules, distal convoluted tubules and connecting tubules and mediates Cl^-^/HCO_3 _^- ^exchange [14,18]. Loss of pendrin does not effect the arterial pH but results in a lower urinary pH [19]. In the inner ear, pendrin is localized in the outer sulcus epithelial cells, root cells, apical membranes of spiral prominence surface epithelial cells and in apical membranes of spindle-shaped cells of stria vascularis [11,17].

A model for Pendred syndrome, consisting of mice lacking functional expression of pendrin, has recently been developed [20]. Similar to patients suffering from Pendred syndrome, *Slc26a4*^-/- ^mice are deaf, have an enlarged vestibular aqueduct and appear to be euthyroid. Mice, in contrast to human patients, do not exhibit goiter. Adult *Slc26a4*^-/- ^mice do not generate an endocochlear potential, which is generated by stria vascularis and is necessary for normal hearing [15,17].

We have shown that adult *Slc26a4*^-/- ^mice show signs of degeneration of stria vascularis, including hyperpigmentation and marginal disorganization [15,17]. It remains unclear, however, whether hyperpigmentation and marginal cell disorganization occurred before or after the normal onset of hearing (P10 or P15, respectively). Further, it remains unclear whether the disorganized surface epithelial cells were all marginal cells or whether different cells rose to the epithelial surface of stria vascularis, giving rise to the disorganized appearance. In the present study, we determined the time course of hyperpigmentation and marginal disorganization. Further, we tested the hypothesis that hyperpigmentation and marginal cell disorganization is a consequence of tissue inflammation including an invasion of inflammatory cells.

## Methods

### Animals

Breeding pairs of *Slc26a4*^-/- ^and *Slc26a4*^+/+ ^mice were obtained from the colony of Dr Susan Wall (Emory University, Atlanta, GA, USA) to establish a new colony at KSU. Mice used for this study were anaesthetized either with 4% tribromoethanol (0.014 ml/g body weight i.p.) or pentobarbitol (0.1 mg/g body weight, i.p.) and sacrificed by decapitation or by transcardial perfusion. Transcardial perfusion consisted of Cl^-^free solution (6 ml, 1 min) followed by Cl^- ^free solution containing 4% paraformaldehyde (24 ml, 4 min). Cl^- ^free solution contained mM 150 mM Na-gluconate, 1.6 mM K_2_HPO_4_, 0.4 mM KH_2_PO_4_, 4 mM Ca-gluconate_2_, 1 mM MgSO_4 _and 5 mM glucose, pH 7.4.

Mice that either express (*Slc26a4*^+/+ ^or *Slc26a4*^+/-^) or lack (*Slc26a4*^-/-^) a functional pendrin gene were studied at four developmental stages, before and after the age that marks the onset of hearing at postnatal day 10 (P10) and P15, respectively, after weaning (P30-41) and adult (P74-170). Genotypes were determined by PCR as described previously [20]. All procedures involving animals were approved by the Institutional Animal Care and Use Committee of Kansas State University.

### Confocal microscopy of cryosections

Temporal bones from age matched *Slc26a4*^-/- ^and *Slc26a4*^+/- ^mice were rendered blood free and fixed by transcardial and perilymphatic perfusion with Cl^- ^free solution containing 4% paraformaldehyde. Temporal bones were decalcified in EDTA, processed through a sucrose gradient and infiltrated with polyethylene glycol. Mid-modiolar cryosections (12 μm, CM3050S, Leica, Nussloch, Germany) were blocked in PBS-TX (137 mM NaCl, 10.1 mM Na_2_HPO_4_, 1.8 mM KH_2_PO_4_, 2.7 mM KCl, pH 7.4 with 0.2% Triton X 100) and 5% bovine serum albumin. Slides were incubated overnight at 4°C with Alexa-488 conjugated rat anti-mouse *CD68 *antibody (1:25, Serotec, Raleigh, NC, USA) in PBS-TX with 1–3% BSA. After incubation, slides were washed with PBS-TX, mounted with FluorSave (Calbiochem, La Jolla, CA, USA), and viewed by confocal and laser scanning brightfield microscopy (LSM 510 Meta, Carl Zeiss, Göttingen, Germany).

### Confocal microscopy of whole-mounts

Temporal bones from age matched *Slc26a4*^-/- ^and *Slc26a4*^+/- ^mice were rendered blood free by transcardial perfusion with Cl^- ^free solution. Stria vascularis was obtained by microdissection and fixed for 2 hrs at 4°C in Cl^- ^free solution containing 4% paraformaldehyde, washed twice in Cl^- ^free solution and once in PBS-TX and then blocked with 5% BSA in PBS- TX for 45 min at RT and then washed three times in PBS-TX. Stria vascularis was then incubated overnight at 4°C either with Alexa-488 conjugated rat anti-mouse *CD68 *antibody (1:25, see above) or goat anti-*Kcnq1 *primary antibody (1:200, C20, Santa Cruz Biotechnology, Santa Cruz, CA, USA) in PBS-TX with 1–3% BSA. Tissues incubated with goat anti-*Kcnq1 *primary antibody were washed with PBS-TX and incubated for 1 h at 25°C with chicken anti-goat Alexa 594 secondary antibody, 1:1,000 (Molecular Probes, Eugene, OR, USA) in PBS-TX with 1–3% BSA.

After antibody incubation, stria vascularis was washed in PBS-TX (0.2% Triton-X) and stained with phalloidin conjugated to Alexa 594 (1:40; Invitrogen, Carlsbad, CA, USA), washed three times with PBS-TX, mounted with FluorSave (Calbiochem), and viewed by confocal and laser scanning brightfield microscopy (LSM 510 Meta, Carl Zeiss).

### RNA isolation

Temporal bones were removed and stria vascularis and spiral ligament were obtained by microdissection. Microdissection solutions were changed twice and isolated tissues were washed to minimize contamination between tissue fractions. In addition, liver and spleen were collected and rapidly frozen in liquid nitrogen. Total RNA was isolated and residual DNA contamination was removed by DNase treatment (RNeasy micro, Qiagen, Valencia, CA, USA). Frozen samples of liver and spleen were pulverized and homogenized. Total RNA was isolated and freed from DNA contamination (RNeasy mini, Qiagen). Isolated RNA was either used immediately or diluted and stored at -80°C for later analysis (RNA storage solution, Ambion, Austin, TX, USA).

### Gene array

Total RNA was isolated from stria vascularis of adult *Slc26a4*^+/+ ^and *Slc26a4*^-/- ^mice (P148 ± 3 and P153 ± 1, respectively). The minimal amount of blood present within capillaries was deemed insignificant; no cardiac perfusion was performed. Isolated RNA was concentrated and frozen for shipment to the Biotechnology Support Facility at University of Kansas Medical Center. A total of six chips were run; each chip was used to analyze RNA pooled from two animals. Three chips each were used to analyze RNA expression in stria vascularis of *Slc26a4*^+/+ ^and *Slc26a4*^-/- ^mice. RNA was amplified by two rounds of amplification, and cRNA was hybridized to high-density oligonucleotide gene chips (Small sample protocol, version II; mouse 430 2.0 gene chip, Affymetrix, Santa Clara, CA, USA). Gene array data were analyzed using commercial software (GCOS, Affymetrix; Genespring, Silicon Genetics, Redwood City, CA, USA) as well as custom-written macros (Excel, Microsoft, Redmond, WA, USA). Quality metrics conformed with MIAME standards (Table 1). Present/absent calls and averaged signal intensities (average of data obtained from three chips) were used to determine expression and changes in expression levels, respectively.

**Table 1:** Quality metrics of gene arrays. Gene array data were deposited at GEO (GSE4749). The required MIAME quality metrics are given as average ± SD.

Chips	Scale Factor	Average call (All)	Present calls (%)	Background intensity	Noise	Raw Q
6	3.05 ± 1.07	682.9 ± 23.6	42.2 ± 3.1	100.3 ± 18.9	5.4 ± 1.1	3.1 ± 0.5

In the tabulated data summaries, 'Intensity' for *Slc26a4*^+/+ ^and for *Slc26a4*^-/- ^samples represents averages of data from one or more probes. For example, the gene *Slc12a2 *is represented on the chip by four probes. Present calls (P) were summarized for all three chips, e.g. 12/12 indicates that this gene was called present by all 12 probes (4 × 3 = 12); 9/15 indicates that the gene is represented by 5 probes on the 3 chips (5 × 3 = 15) and that the gene was called present by 9 of the 15 probes.

Ratios of intensity values (*Slc26a4*^-/- ^to *Slc26a4*^+/+^) were calculated for each probe and averaged. Average ratios > 1.000 were reported as *Fold *with the *Direction *'up'. Average ratios < 1.000 were inverted (1/average ratio) and reported as *Fold *with the *Direction *'down'. *Fold *values are given in the tables only when the gene was called Present (P) in *Slc26a4*^+/- ^or *Slc26a4*^-/- ^samples in at least half of the available probes. The direction of the fold change is only given when it exceeded 1.30. Fold changes lower than 1.30 were not considered significant.

### Quantitative RT-PCR

Age and sex matched *Slc26a4*^-/- ^and *Slc26a4*^+/- ^mice were rendered blood free by transcardiac perfusion with Cl^- ^free solution. Total RNA was isolated from microdissected stria vascularis and spiral ligaments, as well as from liver and spleen. In each 96-well plate, the expression of seven different genes as well as 18S rRNA was analyzed. Total RNA from stria vascularis and spiral ligaments or from spleen and liver of one *Slc26a4*^-/- ^and one matched *Slc26a4*^+/- ^mouse was analyzed in duplicate reactions in parallel to allow paired comparisons (paired t-test). qRT-PCR was performed in the presence of 0.5× SYBR green I on total RNA isolated from individual animals using gene specific primers (One step RT-PCR kit, Qiagen; iCycler, BioRad, Hercules, CA, USA; SYBR green I, Molecular Probes; Table 2). RT was performed for 30 min at 50°C and 15 min at 95°C. PCR consisted of 40 cycles of 1 min at 60°C, 1 min at 72°C, 20 s hot measurement, and 1 min at 94°C. Specificity of primers was verified by sequencing. The generation of a single product of the appropriate size was verified by agarose gel electrophoresis.

**Table 2:** Sequences of gene specific primers

Gene	Primer sequence	Product length (bp)
18S	gag gtt cga aga cga tca ga (sense)	
	tcg ctc cac caa cta aga ac (antisense)	317
*C1r*	aac aag atg ctg ctg acc (sense)	
	tat tca agg ctg gag aca tag (antisense)	302
*C3*	atc cga tac tac acc tacc (sense)	
	cct ggt ttc ctt caa tcc (antisense)	307
*Ptprc (Cd45)*	tgt tga cag agt tag tga atg g (sense)	
	gtg gac gag gat gga tgc (antisense)	323
*Lyzs*	cct gct ttc tgt cac tgc tc (sense)	
	ggt gta atg atg gca aaa cc (antisense)	235
*Il1a*	aca cta tct cag cac cac ttg g (sense)	
	gca ccc gac ttt gtt ctt tgg (antisense)	311
*Il6*	ctg caa gag act tcc atc c (sense)	
	tat atc cag ttt ggt agc atc c (antisense)	300
*Il12a*	tca atc acg cta cct cct c (sense)	
	ctg ttg tgg aag aag tct ctc (antisense)	302
*Nos2*	gtt tga cca gag gac cca ga (sense)	
	acc tga tgt tgc cat tgt tg (antisense)	354
*Nos3*	cct aca gag cag caa atc ca (sense)	
	gcc ttt ctc cag ttg ttc ca (antisense)	202
*Otos*	atg cag ccc tgt ctg ctg tgg tgg (sense)	
	gtc ctc ctg gta ggg aac atg gaa (antisense)	267
*Tyr*	gcc tgt gcc tcc tct aag (sense)	
	ttc taa tca aga ctc gct tct c (antisense)	295

Template molecules were quantified according to T = 10^log (P_Ct_)/(E_avg_^C_t_), where P_Ct _is product molecules at C_t_, E_avg _is the average efficiency and C_t _is cycle at which the fluorescence of the product molecules reached a set threshold. Efficiencies for individual reactions was obtained from the slope of the log-linear phase of the growth curve using an Excel-based program (LinRegPCR) [21].

The number of product molecules at C_t _(P_Ct_) was calculated by amplifying known numbers of 18S rRNA (T_18S_) molecules according to P_Ct _= T_18S _× E_18S_^C_t_, where E_18S _is the average efficiency of all the 18S rRNA reactions. The mass of 18S rRNA in 1 μg of total RNA was estimated to calculate the number of 18S rRNA molecules in a given amount of RNA sample. Calculations were based on three assumptions: firstly, total RNA consists to 100% of 18S and 28S rRNA. Secondly, 18S and 28S rRNA occur in a 1:1 ratio. Thirdly, murine 18S and 28S rRNA contains 1869 and 4712 nucleotides [Genbank: X00686] and [Genbank: X00525], respectively. The mass of 18S rRNA per 1 μg of total RNA was estimated to be 0.284 μg, equivalent to 4.7 × 10^-13 ^mol or 2.8 × 10^11 ^molecules of 18S rRNA (molecular weight of 18S rRNA estimated to be 598,080).

### Western blotting

Temporal bones from age matched *Slc26a4*^-/- ^and *Slc26a4*^+/- ^mice were rendered blood free by transcardial perfusion with Cl^- ^free solution. Stria vascularis and spiral ligament were isolated by microdissection. Proteins in stria vascularis and spiral ligament from one animal were isolated by heating (10 min, 95°C) in 20 μl of a diluent (Compound B, NanoOrange, Invitrogen). After cooling (20 min, RT), the isolated protein was quantified (NanoOrange, Invitrogen) and either used immediately in Western blots or stored at -80°C.

Proteins (15 μg per well) were denatured in the presence of Laemmli sample buffer (62.5 mM Tris-HCl, pH 6.8, 25% glycerol, 2% SDS, 0.01% bromophenol blue, 5% 2-mercaptoethanol) at 80°C for 5 min and resolved by electrophoresis (150 V for 45 min) in 4–15% polyacrymalide Tris-HCl gradient gels (10 well mini-gel, BioRad laboratories, Inc., Hercules, CA, USA). Molecular weight standards were used to estimate molecular weights (Precision Markers, BioRad). Proteins were transferred in transblot buffer (25 mM Tris, 192 mM glycine, 20% v/v methanol, pH 8.3). (25 V, 1 h; XCell II blot module, Invitrogen) onto a nitrocellulose membrane (0.2 μm, BioRad).

Nitrocellulose membranes were first evaluated for lysozyme expression, then stripped (Restore Western Blot Stripping Buffer, Pierce, Rockford, IL, USA) and evaluated for actin expression. Membranes were blocked in TBS-Tween (20 mM Tris-Cl, pH 7.6 with 0.1% Tween) containing 5% non-fat dry milk powder (BioRad) and incubated (1 hr RT) with rabbit anti-human lysozyme primary antibody (1:500; Dakocytomation, Carpinteria, CA) or rabbit anti-Actin primary antibody (1:1,000; Sigma) in TBS-Tween containing 5% non-fat dry milk. Membranes were washed three times with TBS-Tween and then incubated with horseradish peroxidase conjugated donkey anti-rabbit antibody (1:25,000, Amersham Biosciences) in TBS-Tween containing 5% non-fat dry milk. The membranes were washed again three times with TBS-Tween and then treated with chemiluminescent detection agents (SuperSignal West Femto Maximum Sensitivity Substrate; Pierce), exposed to film (CL-XPosure Film, Pierce), which was developed immediately (XOmat 2000A, Kodak, Rochester, NY, USA).

### Statistics

Numeric data are presented at average ± sem, unless specified otherwise. The number (n) of animals, blots or cells is given. Differences were determined by paired t-tests. Significance was assumed at p < 0.05.

## Results and Discussion

### Cellular reorganization of stria vascularis

**Figure 1:**
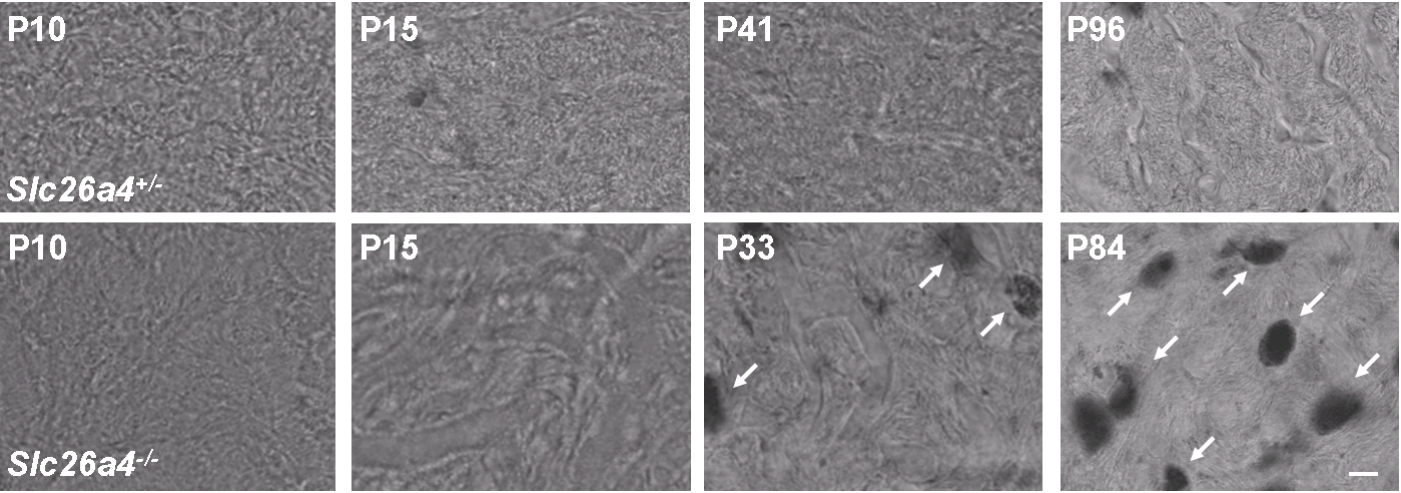
**Hyperpigmentation in stria vascularis**. The time course of hyperpigmention during development was determined by laser scanning brightfield microscopy of stria vascularis whole-mounts. Top: images from *Slc26a4*^+/- ^mice of various ages (P, postnatal day). Bottom: images from *Slc26a4*^-/- ^mice. Note that hyperpigmentation (arrows) was observed in *Slc26a4*^-/- ^mice at P33 and P84 but not at P10 or P15. No hyperpigmentation was observed in *Slc26a4*^+/- ^mice. The scale bar shown in the bottom right image represents 10 μm and pertains to all images in this figure.

Previously, we reported that stria vascularis of adult *Slc26a4*^-/- ^mice is hyperpigmented and that marginal cells appear disorganized [15,17]. Stria vascularis was obtained from age matched *Slc26a4*^+/- ^and *Slc26a4*^-/- ^mice and hyperpigmentation was evaluated by laser-scanning light microscopy. Hyperpigmentation was absent at P10 and P15 but observed after weaning (P33-41) and in adult (P84-96) *Slc26a4*^-/- ^mice (Fig. 1).

**Figure 2:**
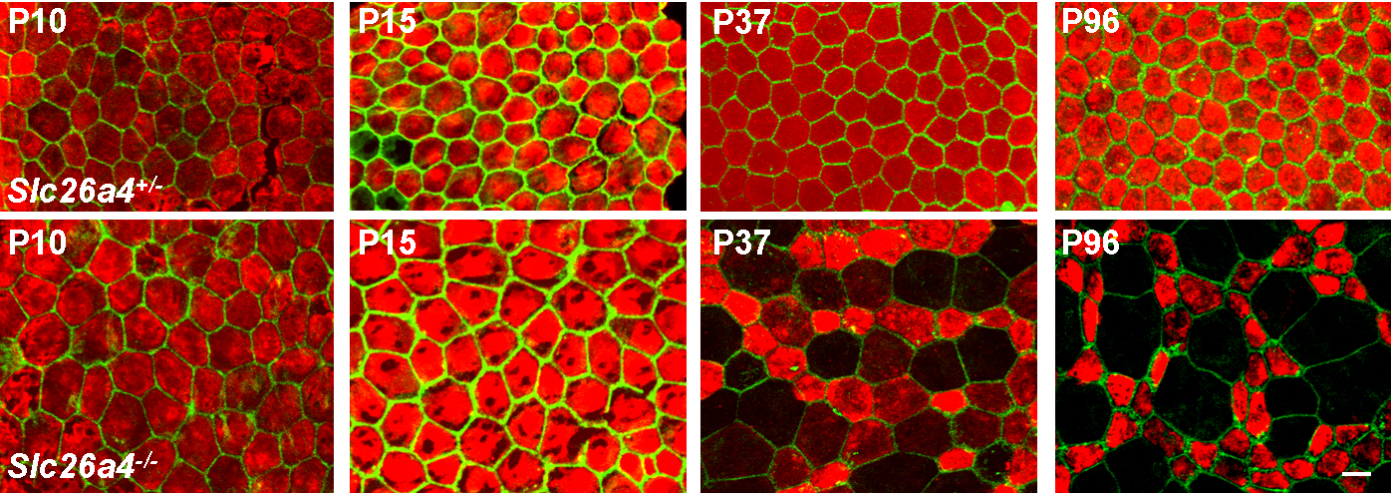
**Loss of Kcnq1 expression in marginal cells**. The time course of marginal cell reorganization during development was determined by confocal immunohistochemistry of *Kcnq1 *(red) and phalloidin-staining of *α-actin *(green) of stria vascularis in whole-mounts. *Top*, images from *Slc26a4*^+/- ^mice of various ages. *Bottom*, images from *Slc26a4*^-/- ^mice. Note that marginal cells of *Slc26a4*^+/- ^mice expressed *Kcnq1 *homogeneously in their apical membrane and that apical membrane surface areas displayed little variation in size. Marginal cells of *Slc26a4*^-/- ^mice at P10 and P15 expressed *Kcnq1 *homogeneously, too, and apical membrane surface areas showed little variation in size. However, surface areas were larger compared to *Slc26a4*^+/- ^mice. At P37, expression of *Kcnq1 *in *Slc26a4*^-/- ^mice was reduced in some marginal cells and maintained in others. Loss of *Kcnq1 *expression correlated with an enlargement of the apical membrane surface area and maintenance of *Kcnq1 *expression correlated with a reduction in the apical membrane surface area. This segregation of marginal cells was more drastic at P96. The scale bar shown in the bottom right image represents 10 μm and pertains to all images in this figure.

**Figure 3:**
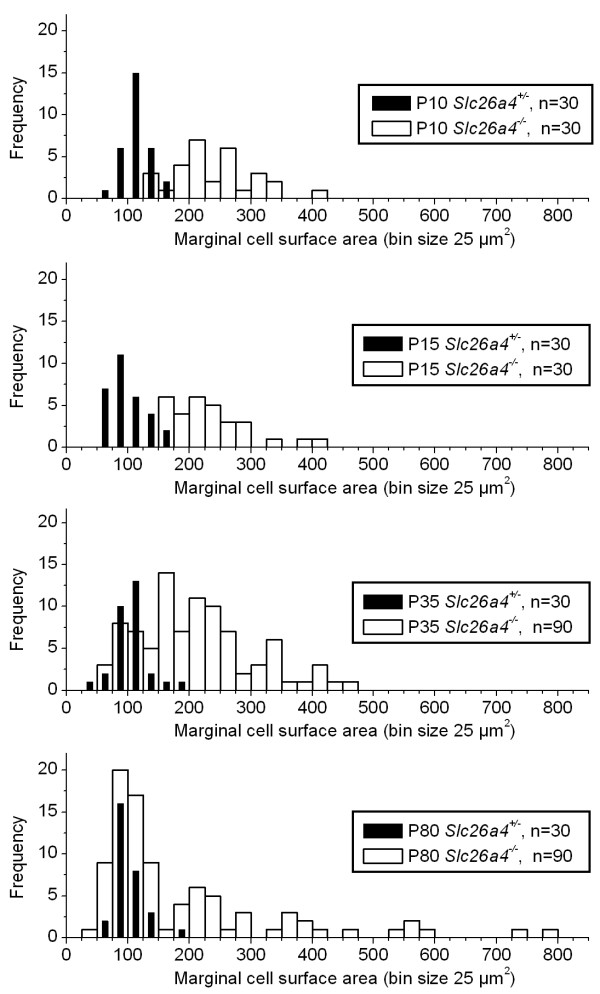
**Reorganization of marginal cells**. Apical surface areas of marginal cells were measured using images of whole-mounts of stria vascularis as shown in Fig. 2. Frequency histograms (bin size 25 μm^2^) of surface area measurements were constructed. The number (n) of cells contributing to each histogram is given. Note that surface areas in *Slc26a4*^+/- ^mice of all ages were normally distributed and narrowly centered at ~ 100 μm^2^. The distribution of surface areas in *Slc26a4*^-/- ^mice progressed during development from a distribution centered at ~ 240 μm^2 ^to a wide multimodal distribution with peaks centered at ~ 100 and ~ 240 μm^2^.

Marginal cells can be identified by there expression of the K^+ ^channel *Kcnq1 *in their apical membrane [17]. To gain information on the identity of surface epithelial cells, expression of *Kcnq1 *was visualized by immunocytochemistry and F-actin, a marker for tight junctions, was visualized by phalloidin staining. Marginal cells at all ages of *Slc26a4*^+/- ^mice and at P10 and P15 of *Slc26a4*^-/- ^mice expressed the K^+ ^channel *Kcnq1 *evenly. Little variation was observed in cell surface areas at all ages of *Slc26a4*^+/- ^mice and at P10 and P15 of *Slc26a4*^-/- ^mice, and variations appeared normally distributed (Figs 2 and 3). The average cell surface area of marginal cells in *Slc26a4*^+/- ^mice was 104 ± 2 μm^2 ^(n = 120). No significant differences were detected in *Slc26a4*^+/- ^mice of different ages. In contrast, the average cell surface areas of marginal cells in P10 and P15 *Slc26a4*^-/- ^mice were significantly larger, 236 ± 12 μm^2 ^(n = 30) and 232 ± 12 μm^2 ^(n = 30), respectively. This enlargement of the apical surface area of marginal cells is consistent with the understanding that *Slc26a4*^+/- ^and *Slc26a4*^-/- ^mice do not differ in the total number of marginal cells, and that cell surface areas are enlarged to cover the larger area that results from the enlarged cochlear diameter found in *Slc26a4*^-/- ^mice.

After weaning, marginal cells of *Slc26a4*^-/- ^mice began to reorganize, leading to a multimodal distribution of cell surface areas (Figs 2 and 3). Most interestingly, marginal cells that reduced their surface areas from ~ 240 to ~ 100 μm^2 ^retained dense expression of *Kcnq1*, whereas marginal cells that increased their surface area gradually lost *Kcnq1 *expression. The degree of *Kcnq1 *staining in stria vascularis of P37 *Slc26a4*^-/- ^mice correlated inversely with the size of the cell surface area. Stria vascularis of adult *Slc26a4*^-/- ^mice consisted of a mosaic of *Kcnq1*-expressing and degenerated marginal cells. *Kcnq1*-expressing marginal cells are likely to remain functional, given that *Kcnq1 *is essential for K^+ ^secretion and the endolymphatic K^+ ^concentration was found to be normal (~ 140 mM) in adult *Slc26a4*^-/- ^mice [17,22,23].

### Expression analysis by gene array

Gene expression in stria vascularis was analyzed by gene array to gain data on the cause of marginal cells reorganization. The quality of the gene array was determined by quality metrics (Table 1), and the quality of the expression analysis was determined by an evaluation of genes that are known to be expressed in stria vascularis (Table 3) and by an evaluation of genes that are known to be expressed in neighboring tissues that could have served as sources of contamination (Table 4). As expected, genes known to be expressed in marginal, intermediate basal and spindle cells were present in *Slc26a4*^+/+ ^and *Slc26a4*^-/- ^mice (Table 3). Interestingly, genes known to be expressed in marginal cells were downregulated in *Slc26a4*^-/- ^mice (Table 3A). In contrast, no change in expression was observed for genes known to be expressed in intermediate and/or basal cells (Table 3B). These data are consistent with a partial degeneration of marginal cells.

**Table 3:** Genes that were expected to be expressed in stria vascularis.

			*Slc26a4*^+/+^		*Slc26a4*^-/-^			
No.	Gene	Description	Intensity	P	Intensity	P	Fold	Direction
1	*Slc12a1*	Na^+^/2Cl^-^/K^+ ^cotransporter	14,664	12/12	10,656	12/12	1.33	down
2	*Atp1a1*	Na^+^/K^+ ^ATPase, alpha	7,276	9/15	5,440	9/15	2.06	down
3	*Atp1b1*	Na^+^/K^+ ^ATPase, beta1	10,593	12/18	8,587	12/18	1.20	
4	*Atp1b2*	Na^+^/K^+ ^ATPase, beta2	8,057	6/6	5,984	6/6	1.41	down
5	*Kcnq1*	K^+ ^channel, alpha	858	15/24	542	11/24	2.48	down
6	*Kcne1*	K^+ ^channel, beta	9,042	6/6	3,490	6/6	2.60	down
7	*Kcnma1*	K^+ ^channel, BK	270	10/18	159	9/18	1.72	down
8	*Kcnk1*	K^+ ^channel	2,667	6/6	1,670	6/6	1.92	down
9	*Slc26a4*	Pendrin	1,136	3/3	619	3/3	1.84	down
10	*Kcnj10*	K^+ ^channel	17,169	3/3	17,005	3/3	1.01	
11	*Car2*	Carbonic anhydrase	9,139	3/3	9,167	3/3	1.00	
12	*Gjb2*	Connexin 26	12,785	3/3	12,629	3/3	1.01	
13	*Gjb6*	Connexin 30	15,257	3/3	13,060	3/3	1.17	
14	*Sod1*	Superoxide dismutase	4,149	14/15	3,538	14/15	1.21	
15	*Cat*	Catalase	3,520	6/6	4,686	6/6	1.12	
16	*Gpx1*	Glutathione peroxidase	2,633	3/3	4,320	3/3	1.64	up
17	*Gpx3*	Glutathione peroxidase	12,526	3/3	9,898	3/3	1.27	
18	*Gpx4*	Glutathione peroxidase	11,224	6/6	10,421	6/6	1.06	
19	*Tyr*	Tyrosinase	3,520	6/6	4,686	6/6	1.16	

Genes that are expected to be expressed in marginal and spindle cells of stria vascularis (1–9) and genes that are expected to be expressed in intermediate and basal cells of stria vascularis (10–19) are listed. For parameters 'P', 'Fold' and 'Direction', see the Gene array section in Methods. Fold changes are given when a gene was called Present (P) in *Slc26a4*^+/- ^or *Slc26a4*^-/- ^samples in at least half of the available probes. The direction of the fold change is only given when it exceeded 1.30. Fold changes lower than 1.30 were not considered significant.

**Table 4:** Genes that were not expected to be expressed in stria vascularis.

			*Slc26a4*^+/+^		*Slc26a4*^-/-^			
No.	Gene	Description	Intensity	P	Intensity	P	Fold	Direction
1	*Pres*	Prestin	53	0/3	8	0/3		
2	*Kcnq4*	K^+ ^channel	160	0/6	160	0/6		
3	*Atp2b2*	PMCA2	35	0/9	116	3/9		
4	*Calb1*	Calbinin 28K	53	0/12	36	0/12		
5	*Calb2*	Calretinin	28	0/3	27	0/3		
6	*Ache*	Acetylcholinesterase	57	0/3	5	0/3		
7	*Slc1a3*	Glutamate transporter GLAST	122	3/18	154	8/18		
8	*Gria1*	Glutamate receptor, ionotropic	57	0/9	55	0/9		
9	*Atp6v1b1*	H^+ ^ATPase	58	0/3	26	0/3		
10	*Tecta*	Tectorin, alpha	62	0/3	52	0/3		
11	*Tectb*	Tectorin, beta	90	0/3	01	0/3		
12	*Otog*	Otogelin	230	0/3	63	0/3		
13	*Otof*	Otoferlin	231	0/3	214	0/3		
14	*Otop1*	Otopetrin 1	54	0/3	62	1/3		
15	*Otop2*	Otopetrin 2	247	0/3	203	0/3		
16	*Otop3*	Otopetrin 3	60	0/3	47	0/3		
17	*Otos*	Otospiralin	636	3/3	2,368	3/3	3.73	up
18	*Slc4a7*	Na^+^/HCO_3 _^- ^cotransporter	398	3/3	444	3/3	1.11	
19	*Kcnj16*	K^+ ^channel	280	2/6	239	1/6		

Genes that are expected to be expressed in the organ of Corti, the modiolus or the vestibular labyrinth but not in stria vascularis (1–16), and genes that are expected to be expressed in spiral ligament but not in stria vascularis (17–19) are listed. For parameters 'P', 'Fold' and 'Direction', see the Gene array section in Methods. Fold changes are given when a gene was called Present (P) in *Slc26a4*^+/- ^or *Slc26a4*^-/- ^samples in at least half of the available probes. The direction of the fold change is only given when it exceeded 1.30. Fold changes lower than 1.30 were not considered significant.

Genes known to be expressed in neighboring tissues were not found in stria vascularis, with the exception of otospiralin (*Otos*). *Otos *in known to be expressed in spiral ligament [24], which is the tissue adjacent to stria vascularis (Table 4). Taken together, these data demonstrate that gene array analysis provided reliable data of expression in stria vascularis.

### Macrophage invasion of stria vascularis

Gene array analysis revealed that stria vascularis of adult *Slc26a4*^-/- ^mice expresses markers specific and/or consistent with the presence of macrophages (Table 5). Expression of *Mac2 *antigen, *Itgax*, *Cd45*, *Cd83 *and *Cd68 *is limited to leucocytes and hematopoietic cells including macrophages and dendritic cells [25]. Expression of major histocompatibility complex II (MHCII) proteins is limited to antigen-presenting cells including macrophages [25]. The expression of *Lysz*, a lysosomal enzyme, has been shown to be a marker for monocytes and macrophages [26]. Further, the increased expression of major histocompatibility complex I, of complement components and of cathepsins is consistent with the presence of macrophages although the expression of the gene is not limited to macrophages [27].

**Table 5:** Markers for macrophages/dendritic cells.

			*Slc26a4*^+/+^		*Slc26a4*^-/-^			
No.	Gene	Description	Intensity	P	Intensity	P	Fold	Direction
1	*Ptprc*	Cd45 antigen	226	3/3	946	3/3	4.19	up
2	*Cd83*	Cd83 antigen	126	0/3	524	3/3	4.16	up
3	*Cd68*	Cd68 antigen	170	3/3	1,344	3/3	7.90	up
4	*Cd14*	Cd14 antigen	412	1/3	619	3/3	1.50	up
5	*H2*-*Aa*	MHC class II	264	5/12	1,019	9/12	4.00	up
6	*H*2-*Ab*	MHC class II	126	0/9	279	3/9	1.86	up
7	*H2*-*Eb1*	MHC class II	429	0/3	1,010	3/3	2.35	up
8	*Lgals3*	Mac2 antigen	91	2/6	1,866	5/6	13.49	up
9	*Itgax*	Integrin alpha X	125	0/3	375	3/3	3.00	up
10	*Emr1*	Mucin receptor	750	3/3	1,547	3/3	2.06	up
11	*Lyzs*	Lysozyme	551	6/9	8257	9/9	11.78	up
12	*H2*-*K1*	MHC class I (D-region)	1,607	13/18	4,062	15/18	2.06	up
13	*H2*-*K1*	MHC class I (K-region)	1,828	6/12	2,883	6/12	1.54	up
14	*H2*-*K1*	MHC class I (Q-region)	549	1/3	890	3/3	1.62	up
15	*C1r*	Complement	37	2/6	266	6/6	13.87	up
16	*C1s*	Complement	71	0/3	200	1/3		
17	*C1qa*	Complement	355	1/3	2,905	3/3	8.19	up
18	*C1qb*	Complement	552	5/6	2,001	6/6	4.43	up
19	*C2*	Complement	3,207	9/9	4,794	6/6	1.62	up
20	*C3*	Complement	11	0/3	231	2/3	20.68	up
21	*C4*	Complement	1,556	3/3	4,788	3/3	3.08	up
22	*C3ar1*	Complement receptor	177	3/9	482	6/9	2.93	up
23	*Ctss*	Cathepsin S	2,304	3/3	7,394	3/3	3.21	up
24	*Ctsk*	Cathepsin K	170	1/3	377	3/3	2.21	up
25	*Ctsb*	Cathepsin B	4,172	12/15	6,576	12/15	1.90	up
26	*Msr2*	Macrophage scavenger receptor	169	3/3	1140	3/3	6.75	up
27	*Mpeg1*	Macrophage expressed gene	582	0/3	5,527	3/3	9.49	up
28	*Cst3*	Cystatin C	5,562	3/3	8,652	3/3	1.56	up
29	*Fcgr1*	IgG receptor, high affinity	98	0/6	163	5/6	1.70	up
30	*Fcgr2b*	IgG receptor, low affinity	112	2/15	377	7/15	3.94	up

Genes that can serve as markers macrophages/dendritic cells (1–10) and genes that are consistent with the presence of macrophages/dendritic cells (11–27) are listed. For parameters 'P', 'Fold' and 'Direction', see the Gene array section in Methods. Fold changes are given when a gene was called Present (P) in *Slc26a4*^+/- ^or *Slc26a4*^-/- ^samples in at least half of the available probes. The direction of the fold change is only given when it exceeded 1.30. Fold changes lower than 1.30 were not considered significant.

Taken together, the data presented in Table 5 suggest that stria vascularis of adult *Slc26a4*^-/- ^mice is invaded by macrophages. Markers for T-cells, NK-cells, B-cells, neutrophils and for acute inflammation were absent in stria vascularis of *Slc26a4*^+/+ ^and *Slc26a4*^-/- ^mice (Tables 6 and 7). The absence of markers of acute inflammation was verified by qRT-PCR of total RNA isolated from stria vascularis and spiral ligament of age and sex matched *Slc26a4*^-/- ^and *Slc26a4*^+/- ^mice. No change in expression was found for *Il1a*, *Il6*, *Il12a*, *Nos2 *and *Nos3 *at P15 or at P34 (each n = 4–5; *data not shown*). Thus, no evidence for acute inflammation was obtained. It is conceivable, however, that short periods of acute inflammation escaped detection. Alternatively, it is conceivable that macrophage invasion was not preceded by neutrophil invasion and that macrophage recruitment occurred neutrophil-independently [28]. The source of chemoattractants for monocytes (MCPs) in neutrophil-independent recruitment are endogenously harbored tissue macrophages and mesothelial cells [28]. Such a mechanism could be present in stria vascularis of *Slc26a4*^-/-^mice as the cochlea endogenously harbors macrophages in the fibrocytes of the lateral wall [29]. The mechanism of recruitment into stria vascularis, however, remains unclear.

**Table 6:** Markers for T, NK and B-cells and Neutrophils.

			*Slc26a4*^+/+^		*Slc26a4*^-/-^			
No.	Gene	Description	Intensity	P	Intensity	P	Fold	Direction
1	*Tcra*	T-cell receptor, alpha	214	0/9	190	0/9		
2	*Tcrb*-*V13*	T-cell receptor, beta V13	136	4/36	109	4/36		
3	*Tcrb*-*J*	T-cell receptor, beta V13	71	2/15	50	1/15		
4	*Tcrg*	T-cell receptor, gamma V4	54	0/15	43	0/15		
5	*Cd3d*	Cd3 antigen	10	0/3	8	0/3		
6	*Cd3e*	Cd3 antigen	139	0/6	74	0/6		
7	*Cd3g*	Cd3 antigen	43	0/3	28	0/3		
8	*Cd3z*	Cd3 antigen	182	4/24	139	3/24		
9	*Cd4*	Cd4 antigen	20	0/6	149	0/6		
10	*Cd8a*	Cd8 antigen	144	4/15	143	4/15		
11	*Cd8b*	Cd8 antigen	1,313	3/6	1,343	3/6		
12	*Ncam1*	Cd56	562	8/21	654	12/21	1.16	
13	*Fcgr3*	IgG receptor, low aff, Cd16	162	2/3	401	3/3	2.48	up
14	*Sdc1*	Syndecan 1 (Cd138)	77	3/12	72	3/12		
15	*Cd19*	Cd19 antigen	15	0/3	13	0/3		
16	*Mpo*	Myeloperoxidase	159	0/3	73	0/3		
17	*Defb1*	Beta-defensin 1	66	1/6	72	2/6		
18	*Defb2*	Beta-defensin 2	23	0/3	14	0/3		
19	*Defb3*	Beta-defensin 3	31	0/3	70	0/3		
20	De*f*b4	Beta-defensin 4	85	0/3	63	0/3		
21	*Camp*	Cathelicidin	11	0/3	10	0/3		

Genes that can serve as markers for T-cells (1–11), NK-cells (12–13), B-cells (14–15) and neutrophils (16–21) are listed. For parameters 'P', 'Fold' and 'Direction', see the Gene array section in Methods. Fold changes are given when a gene was called Present (P) in *Slc26a4*^+/- ^or *Slc26a4*^-/- ^samples in at least half of the available probes. The direction of the fold change is only given when it exceeded 1.30. Fold changes lower than 1.30 were not considered significant.

**Table 7:** Genes that serve as markers for acute inflammation.

			*Slc26a4*^+/+^		*Slc26a4*^-/-^			
No.	Gene	Description	Intensity	P	Intensity	P	Fold	Direction
1	*Il1a*	Interleukin 1a	29	0/3	44	0/3		
2	*Il1b*	Interleukin 1b	173	0/3	156	0/3		
3	*Il6*	Interleukin 6	14	0/3	31	0/3		
4	*Il12a*	Interleukin 12a	126	0/3	96	0/3		
5	*Il12b*	Interleukin 12b	70	1/6	73	1/6		
6	*Il10*	Interleukin 10	116	3/3	87	3/3		
7	*Ifng*	Interferon, gamma	71	1/3	24	0/3		
8	*Tnf*	Tumor necrosis factor, alpha	54	0/3	50	0/3		
9	*Nos2*	Nitric oxide synthase, inducible	19	0/3	27	0/3		

Genes that can serve as markers for acute inflammation (1–9) are listed. For parameters 'P', 'Fold' and 'Direction', see the Gene array section in Methods. Fold changes are given when a gene was called Present (P) in *Slc26a4*^+/- ^or *Slc26a4*^-/- ^stria vascularis in at least half of the available probes. The direction of the fold change is only given when it exceeded 1.30. Fold changes lower than 1.30 were not considered significant.

### Macrophage invasion is restricted to stria vascularis

The observation that stria vascularis of adult mice is invaded by macrophages raises the question whether macrophage invasion is systemic and hence found in other organs, such as spleen and liver, or whether macrophage invasion is restricted to stria vascularis and hence not seen in the adjacent tissue, spiral ligament. This is an important issue, as the inner ear is immunologically responsive to systemic infections [30].

Total RNA was isolated from stria vascularis, spiral ligament, liver and spleen from age and sex matched *Slc26a4*^-/- ^and *Slc26a4*^+/- ^mice. The expression of a select group of transcripts was evaluated by qRT-PCR. Possible contamination between stria vascularis and the adjacent spiral ligament was evaluated by quantifying the expression of *Otos *and tyrosinase (*Tyr*) under the assumption that *Tyr *is expressed in stria vascularis and not in spiral ligament, and *Otos *in spiral ligament and not in stria vascularis. The detected expression level of *Tyr *in stria vascularis of *Slc26a4*^+/- ^and *Slc26a4*^-/- ^mice was 120 ± 33 and 50 ± 13 times higher than in spiral ligament, respectively (n = 6) and the detected expression of *Otos *in spiral ligament of *Slc26a4*^+/- ^and *Slc26a4*^-/- ^mice was 108 ± 32 and 50 ± 28 times higher than in stria vascularis, respectively (n = 3). These data illustrate a small contamination between stria vascularis and spiral ligament. Contamination was larger in *Slc26a4*^-/- ^mice (2%) than in *Slc26a4*^+/- ^mice (1%), consistent with the observation during microdissection that stria vascularis from *Slc26a4*^-/- ^is more fragile.

**Figure 4:**
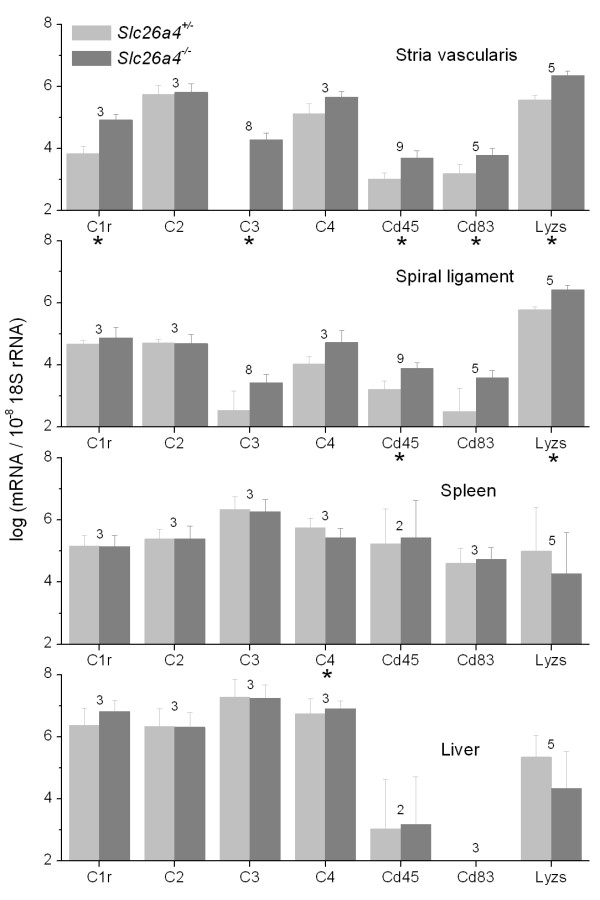
**Tissue specificity of macrophage invasion**. Transcripts of markers specific for or consistent with the presence of macrophages in stria vascularis, spiral ligament, spleen and liver of *Slc26a4*^+/- ^and *Slc26a4*^-/- ^mice at P34 and/or P86 were quantified by qRT-PCR. Significant changes between *Slc26a4*^+/- ^and *Slc26a4*^-/- ^mice are marked with an asterisk (*). Numbers between bars represent the number of animal pairs analyzed. Note that significant increases were mainly seen in stria vascularis, to a lesser degree in spiral ligament, but not in spleen or liver. These data suggest that macrophage invasion is specific to stria vascularis.

Expression of complement components and macrophage markers including *C1r*, *C3*, *Ptprc *(= *Cd45*), *Cd83 *and *Lyzs *was increased in stria vascularis of *Slc26a4*^-/- ^mice compared to *Slc26a4*^+/- ^mice (Fig. 4). Expression of these genes was not upregulated in spiral ligament with the exception of *Lyzs *and *Cd45*. Further, the expression of these genes was not upregulated in liver or spleen (Fig. 4). Taken together, these data suggest that macrophage invasion in *Slc26a4*^-/- ^mice is restricted to stria vascularis.

### Time course of macrophage invasion

**Figure 5:**
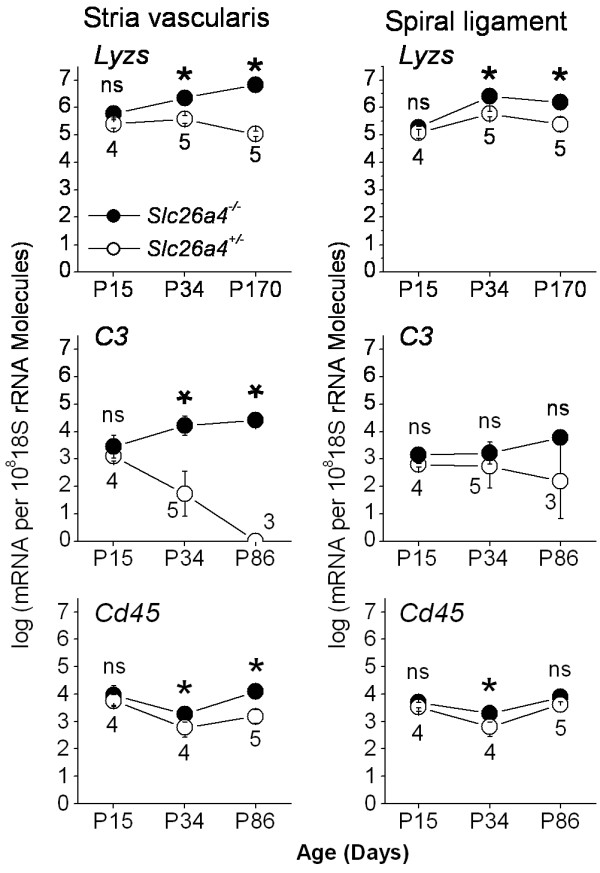
**Time course of macrophage invasion**. Transcripts of markers specific for or consistent with the presence of macrophages in stria vascularis and spiral ligament of *Slc26a4*^+/- ^and *Slc26a4*^-/- ^mice at different ages were quantified by qRT-PCR to determine the time course of macrophage invasion during development. Significant changes between *Slc26a4*^+/- ^and *Slc26a4*^-/- ^mice are marked with an asterisk (*). Numbers between symbols represent the number of age-matched animal pairs analyzed. Note that significant differences were not seen before P34, suggesting that macrophage invasion occurred after weaning (P22).

The finding that macrophage invasion was restricted to stria vascularis raises the question of when in development macrophage invasion occurs. Total RNA was isolated from stria vascularis and spiral ligament from sex and age matched *Slc26a4*^-/- ^and *Slc26a4*^+/- ^mice and a select group of transcripts was quantified by qRT-PCR. At P15, no difference in the expression of *Lyzs*, *C3 *and *Cd45 *was found in stria vascularis or in spiral ligament between *Slc26a4*^-/- ^and *Slc26a4*^+/- ^mice (Fig. 5). After weaning, and persistent into adulthood, however, the expression of *Lyzs*, *C3 *and *Cd45 *was higher in stria vascularis of *Slc26a4*^-/- ^mice compared to *Slc26a4*^+/- ^mice. A similar trend was observed with spiral ligament.

**Figure 6:**
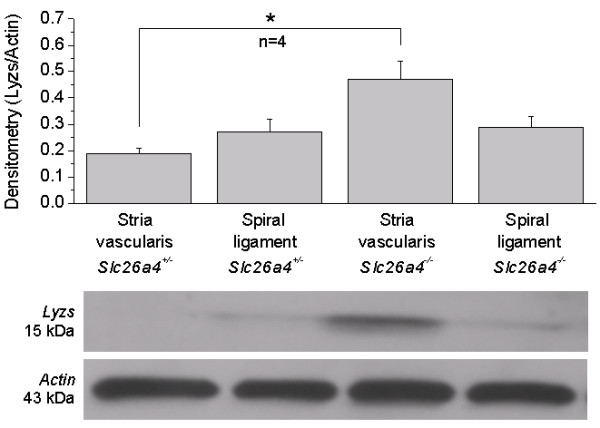
**Lysozyme protein expression**. Protein expression of lysozyme (*Lyzs*) in stria vascularis and spiral ligament of post-weaning *Slc26a4*^+/- ^and *Slc26a4*^-/- ^mice was determined by Western blotting. Actin expression served as a normalization control. Significant changes between *Slc26a4*^+/- ^and *Slc26a4*^-/- ^mice are marked with an asterisk (*). The number of animal pairs (n) is given. Note that protein expression of *Lyzs *was significantly increased in stria vascularis, but not in spiral ligament.

The expression of *Lyzs *was determined based not only on the transcript level but also on the protein level, which may be less prone to contamination between neighboring tissues. Western blotting revealed that *Lyzs *protein is upregulated in stria vascularis but not in spiral ligament of post-weaning *Slc26a4*^-/- ^mice (Fig. 6).

**Figure 7:**
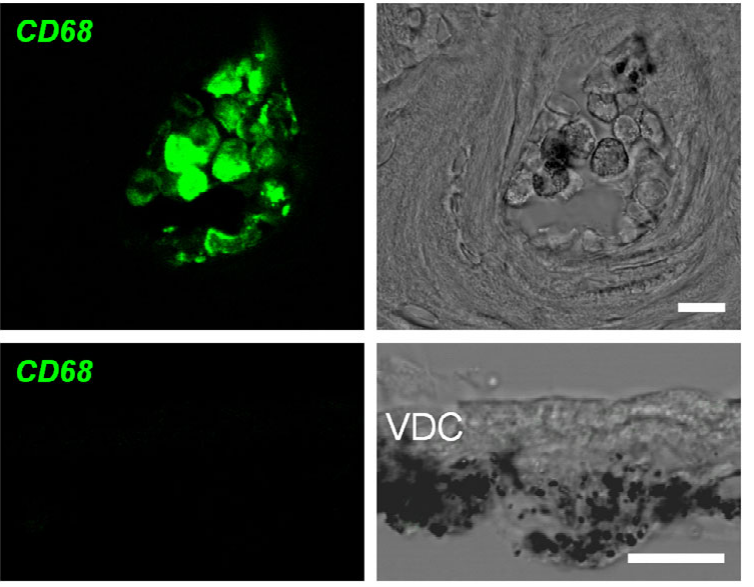
**Controls for immunohistochemistry**. Controls for *CD68 *immunohistochemistry were obtained from cryosections of the cochlea. Top: bone marrow cells congregated in cavities of the bony cochlear wall served as a positive control. Bottom: heavily pigmented cells in the connective tissue underneath vestibular dark cells (VDC) served as a negative control. Left: confocal immunohistochemistry of *CD68*. Right: corresponding bright field images. Scale bars represent 10 μm.

Macrophage invasion was not only determined by gene expression analysis but also by immunohistochemistry, using *CD68 *as a marker. The specificity of the anti-*CD68 *antibody was verified by using bone marrow cells as a positive control and heavily pigmented cells of the vestibular labyrinth as a negative control (Fig. 7). Staining for *CD68 *was clearly associated with hyperpigmentation in stria vascularis in post-weaning and adult *Slc26a4*^-/- ^mice (Fig. 7).

## Conclusion

The data demonstrate that hyperpigmentation of stria vascularis and marginal cell reorganization in *Slc26a4*^-/- ^mice occur after weaning, coinciding with an invasion of macrophages. The data suggest that macrophage invasion contributes to tissue degeneration in stria vascularis, and that macrophage invasion is restricted to stria vascularis and is not systemic in nature. The delayed onset of degeneration of stria vascularis suggests that a window of opportunity exists to restore/preserve hearing in mice and humans suffering from Pendred syndrome.

## Competing interests

The author(s) declare that they have no competing interests.

## Authors' contributions

SVJ and AO drafted the text and PW finalized the manuscript. SVJ carried out confocal immunocytochemistry and morphometry. SVJ and PW isolated tissue fractions by microdissection. AO designed primers, isolated RNA and performed quantitative RT-PCR. RS prepared gene array data for submission under MIAME standards. RJM carried out confocal immunocytochemistry and Western blotting, SF advised on immunology. PW mined gene array data. SMW, LAE and EDG provided mice prior to the establishment of a colony at KSU. PW conceived the study. All authors have read and approved the final manuscript.

**Figure 8:**
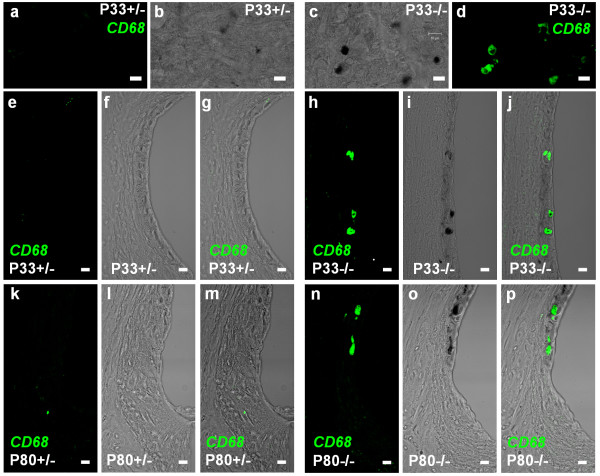
**Macrophage invasion in stria vascularis**. Macrophages were visualized by *CD68 *immunohistochemistry in whole mounts of stria vascularis and cryosections of the cochlear lateral wall. Top row: (a-d), *CD68 *staining in whole mounts of stria vascularis from P33 *Slc26a4*^+/- ^and *Slc26a4*^-/- ^mice. Immunostaining of *CD68 *(a and d) and corresponding bright field images (b and c) are shown. Second and third row: (e-p), *CD68 *staining in cryosections of the cochlear lateral wall from P33 and P80 *Slc26a4*^+/- ^and *Slc26a4*^-/- ^mice. Immunostaining of *CD68 *(e, h, k, and n), corresponding bright field images (f, i, l, and o) and merged images (g, j, m, and p) are shown. Note that *CD68 *expression is restricted to hyperpigmented areas of stria vascularis in *Slc26a4*^-/- ^mice and that no expression of *CD68 *was observed in *Slc26a4*^+/- ^mice. Scale bars represent 10 μm.

## Pre-publication history

The pre-publication history for this paper can be accessed here:


